# Random Finite Set Based Bayesian Filtering with OpenCL in a Heterogeneous Platform

**DOI:** 10.3390/s17040843

**Published:** 2017-04-12

**Authors:** Biao Hu, Uzair Sharif, Rajat Koner, Guang Chen, Kai Huang, Feihu Zhang, Walter Stechele, Alois Knoll

**Affiliations:** 1 Robotics and Embedded Systems, Technische Universität München, 80333 München, Germany; hub@in.tum.de (B.H.); uzair.sharif@tum.de (U.S.); rajat.koner@tum.de (R.K.); guang@in.tum.de (G.C.); knoll@in.tum.de (A.K.); 2 School of Data and Computer Science, Sun Yat-sen University, Xiaoguwei Island, Panyu District, Guangzhou 510006, China; 3 School of Marine Science and Technology, Northwestern Polytechnical University, 127, Youyi West Road, Xi’an 710072, China; feihu.zhang@nwpu.edu.cn; 4 Integrated Systems, Technische Universität München, 80333 München, Germany; walter.stechele@tum.de

**Keywords:** random finite set Bayesian filtering, OpenCL, real-time execution

## Abstract

While most filtering approaches based on random finite sets have focused on improving performance, in this paper, we argue that computation times are very important in order to enable real-time applications such as pedestrian detection. Towards this goal, this paper investigates the use of OpenCL to accelerate the computation of random finite set-based Bayesian filtering in a heterogeneous system. In detail, we developed an efficient and fully-functional pedestrian-tracking system implementation, which can run under real-time constraints, meanwhile offering decent tracking accuracy. An extensive evaluation analysis was carried out to ensure the fulfillment of sufficient accuracy requirements. This was followed by extensive profiling analysis to spot the potential bottlenecks in terms of execution performance, which were then targeted to come up with an OpenCL accelerated application. Video-throughput improvements from roughly 15 fps to 100 fps (6×) were observed on average while processing typical MOT benchmark videos. Moreover, the worst-case frame processing yielded an 18× advantage from nearly 2 fps to 36 fps, thereby comfortably meeting the real-time constraints. Our implementation is released as open-source code.

## 1. Introduction

Current success in the practical implementations of random finite set (RFS) filters has made it clear that RFS-based approaches are going to play a key role in the multisensor data fusion. This is mostly due to the probability hypothesis density (PHD) filters that present a recursive algorithm to jointly estimate target states in the presence of data association uncertainty, detection uncertainty, noise and false alarms [1]. Since then, the performance of new extensions has been increasing at a remarkable pace. The driving force behind the ever-increasing interest in RFS is its high potential in the applications of aerospace, robotics and intelligent systems, as presented in an excellent survey on the multisensor data fusion [2].

While the performance of RFS has been greatly improved, when dealing with real-world applications, running times become important. New computer technologies have already been proposed to accelerate the computation in many machine learning algorithms, but seldom applied to data fusion algorithms. An emerging computing architecture that has been adopted in industry is the heterogeneous system architecture (HSA). HSA is a hardware platform that integrates central processing units, graphics processors or other processors (e.g., FPGA, DSP) on the same bus, with shared memory and tasks. With the support of the Open Computing Language (OpenCL), the HSA is able to relieve the programmer of the task of planning the moving of data between devices’ disjoint memories, thus reducing the mutual communication latency and speeding up the computation.

In this paper, we investigate the problem of applying RFS filtering approaches to a heterogeneous platform, aiming to provide some insights on how to improve the RFS filtering running times by the heterogeneous system. For this purpose, we chose recently-proposed RFS-based filtering techniques i.e., PHD, labeled multi-Bernoulli (LMB) filters, to tackle the underlying multi-target tracking problem. PHD and LMB filters have been presented to be effective in tracking applications that require particle implementation or object individual existence probabilities. However, implementing the tracking algorithm in the heterogeneous system with OpenCL is a non-trivial problem. On the one hand, the tracking application by PHD or LMB filters is a complex and convoluted system that is intrinsically not suitable for parallel execution, as the parallel execution requires the system to be modular, and the execution within each module is independent. On the other hand, to efficiently use OpenCL to accelerate the execution, the execution bottlenecks should be spotted, and OpenCL configurations, such as running batch size or memory usage, should be well tuned. Last, the accuracy should be guaranteed while deploying OpenCL acceleration.

To this end, the tracking algorithms of this paper were developed in a highly modular system design approach and practically implemented in tracking pedestrians of a video. Specifically, we explored the use of the GM-PHD (Gaussian mixture-PHD) and SMC-LMB (sequential Monte Carlo-LMB) class of filters from this family, to implement the tracking algorithm. Initially, pure software (relying solely on CPU code in C++) implementations were carried out following a highly modular overall system-design approach. In order to parallelize the executions as much as possible, we made two major implementation modifications compared to the the original GM-PHD or SMC-LMB. First, we did manual vectorization of code, as GM-PHD uses many small dimension matrices, which are not effective for large-scale parallelization. Second, we consider each Gaussian component independent of each other and splitting them into individual threads. Later, via extensive evaluation analysis concerning tracking accuracy, the GM-PHD trackers were found to be inadequate, and hence, the implementation focus was then shifted solely to SMC-LMB filters. To run the whole tracking application meeting real-time constraints, we carried out an extensive execution profiling of the algorithm. The spotted performance bottlenecks were then ported to the GPU accelerator using OpenCL programming constructs to perform the parallelization potential of the algorithm. We demonstrated the effectiveness of our developed approach by running the MOT benchmark [3]. In particular, we managed to improve from 15 fps to 100 fps in processing MOT video frames on average, while for the most computationally-expensive frame, we achieved 18× speedup improvements from 2 fps to 36 fps. We should note that our SMC-LBM implementation is based on the approach of [4] that separated the prediction and updating in the filtering. Now, another effective approach has been proposed to integrate the prediction and updating together so that the execution would be faster [5]. The discussion of them is in Section 4.

To the best of our knowledge, this is the first paper that reported the implementation results of the RFS-based filtering in a heterogeneous system with OpenCL. Moreover, since the implementation is designed in a highly modular way and the interface of each module is explicit, our developed software is able to be well integrated with other projects. The related open source code is released here (https://github.com/nucleusbiao/Pedestrian-Tracking-using-SMC-LMB-with-OpenCL.git).

This paper is structured as follows: Section 2 describes the mathematic model of GM-PHD filter, as well as the brief introduction of SMC-LMB. Section 3 presents the details of system implementation design. Section 4 presents the simulation and execution results. The paper is concluded in Section 5.

## 2. Preliminary

In the multi-target tracking problem, the number of targets to be tracked is unknown a priori and stochastically varies with time. At the sensor, a random number of measurements is received due to detection uncertainty and false alarms. Consequently, standard Bayesian filtering techniques are not directly applicable, since it is not known which of the received measurements, if any, should be used to update which target state, if any, at each sensor scan.

Towards this problem, the RFS approach is an emerging and promising alternative to the traditional association-based methods like joint probabilistic data association [6] and multiple hypothesis tracking filters [7]. Pioneered by Mahler [8,9], finite-set statistics analysis can be considered as the first systematic and rigorous approach to Bayesian state-estimation while explicitly avoiding the need for cumbersome association of measurements with targets or tracks. The RFS-based filtering enables the use of the optimal Bayesian estimation framework for multi-target tracking scenarios by introducing the concepts of a multi-target state/measurement expressed via random finite sets. In the following, we provide the mathematic background for the Gaussian-mixture PHD filter and briefly introduce the labeled multi-Bernoulli.

### 2.1. Gaussian Mixture-Probability Hypothesis Density Filter

In a multi-target tracking scenario, suppose that, at time $t_{k-1}$, there were M(k−1) targets having states $\left(x_{k-1}^{1},x_{k-1}^{2},...,x_{k-1}^{M(k-1)}\right)\;\mathrm{with}\;\mathrm{each}\;x_{k-1}^{i}\in X$. At $t_{k}$, some of these targets may die; the surviving targets evolve to their new states; and new targets may appear. This results in M(k) targets having new states as $\left(x_{k}^{1},x_{k}^{2},...,x_{k}^{M(k)}\right)$. Similarly, at the sensor, suppose that N(k) measurements $\left(z_{k}^{1},z_{k}^{2},...,z_{k}^{N(k)}\right)\;\mathrm{where}\;\mathrm{each}\;z_{k}^{i}\in Z$ are received at $t_{k}$. States and measurements at $t_{k}$ can be aptly represented as finite sets, as shown below:

(1) $$\begin{array}{cc} X_{k} & =\left\{x_{k}^{1},x_{k}^{2},...,x_{k}^{M(k)}\right\}\in F\left(X\right) \end{array}$$

(2) $$\begin{array}{cc} Z_{k} & =\left\{z_{k}^{1},z_{k}^{2},...,z_{k}^{N(k)}\right\}\in F\left(Z\right) \end{array}$$

where F(X) and F(Z) denote the sets of all finite subsets of X and Z, respectively.

For a given multi-target state $X_{k-1}$ at $t_{k-1}$, each $x_{k-1}\in X_{k-1}$ either continues to exist at $t_{k}$ with survival probability $p_{S,k}\left(x_{k-1}\right)$ or dies with probability $\left(1-p_{S,k}\left(x_{k-1}\right)\right)$. Consequently, for a given state $x_{k-1}$ at $t_{k-1}$, its behavior at the next time step $t_{k}$ is modeled as being an RFS $S_{k|k-1}\left(x_{k-1}\right)$ that can take on either $\left\{x_{k}\right\}$ when the target survives or ϕ when the target dies. A new target born at $t_{k}$ is similarly modeled by an RFS $\Gamma_{k}$. Using these quantities, we can express the multi-target state $X_{k}$ at $t_{k}$ to be:

(3) $$X_{k}=\left(\underset{\zeta \in X_{k-1}}{⋃}S_{k|k-1}\left(\zeta \right)\right)\cup \Gamma_{k}$$

A given target $x_{k}\in X_{k}$ at $t_{k}$ is either detected with detection probability $p_{D,k}\left(x_{k}\right)$ or missed with probability $\left(1-p_{D,k}\left(x_{k}\right)\right)$. Consequently, at each $t_{k}$, each state $x_{k}$ generates a measurement RFS $\Theta_{k}\left(x_{k}\right)$ that can take on either $\left\{z_{k}\right\}$ when the target is detected, or ϕ when the target is missed by the sensor. In addition to these target-originated measurements, the sensor also receives a set of clutter measurements modeled via $K_{k}$ RFS. Thus, given a multi-target state $X_{k}$ at $t_{k}$, the multi-target measurement $Z_{k}$ can be written as:

(4) $$Z_{k}=\left(\underset{x\in X_{k}}{⋃}\Theta_{k}\left(x\right)\right)\cup K_{k}$$

In a similar fashion to STTBayes filtering, the multi-target state-transition densities $p(X_{k}|X_{k-1})$ and the multi-likelihood function $\left(p\left(Z_{k}|X_{k}\right)\right)$ can be derived from the underlying physical models of targets and sensors using FISSTtechniques. Assuming their availability, the multi-target Bayes filter propagates the multi-target posterior state-conditional density $p(Xk|Z^{k})$ via the familiar prediction-update mechanism as follows:

(5) $$\begin{array}{cc} p(X_{k}|Z^{k-1}) & =\int p\left(X_{k}|X\right)p\left(X_{k-1}|Z^{k-1}\right)\delta X \end{array}$$

(6) $$\begin{array}{cc} p(X_{k}|Z^{k}) & =\frac{p\left(Z_{k}|X_{k}\right)p\left(X_{k}|Z^{k-1}\right)}{\int p\left(Z_{k}|X_{k}\right)p\left(X_{k}|Z^{k-1}\right)\delta X} \end{array}$$

where the integrals in the recursion are FISST set integrals as introduced earlier.

To derive the GM-PHD recursion, the multi-target tracking context must also satisfy:

- Each target follows a linear Gaussian dynamical model, and the sensor has a linear Gaussian measurement model:
  (7) $$\begin{array}{cc} p(x|\zeta ) & =N(x;F_{k}\zeta ,Q_{k}) \end{array}$$
  (8) $$\begin{array}{cc} p(z|x) & =N(z;H_{k}x,R_{k}) \end{array}$$
- The survival and detection probabilities are state independent:
  (9) $$\begin{array}{cc} p_{S,k}\left(x\right) & =p_{S,k} \end{array}$$
  (10) $$\begin{array}{cc} p_{D,k}\left(x\right) & =p_{D,k} \end{array}$$
- The PHD or the intensity function of birth RFS $\gamma_{k}$ is a Gaussian mixture (GM) of the form:
  (11) $$\gamma_{k}\left(x\right)=\sum_{i=1}^{J_{\Gamma ,k}}w_{\Gamma ,k}^{i}N\left(x;m_{\Gamma ,k}^{i},P_{\Gamma ,k}^{i}\right)$$

Under these assumptions, it has been shown in [1] that the predicted posterior PHD, as well the posterior state PHD at any $t_{k}$ is also a Gaussian mixture. Specifically, at $t_{k}$, if the prior PHD is expressed as a GM of the form:

(12) $$v_{k-1}\left(x\right)=\sum_{i=1}^{J_{k-1}}w_{k-1}^{i}N\left(x;m_{k-1}^{i},P_{k-1}^{i}\right)$$

then the GM-PHD recursion can be given by:

- Prediction:
  (13) $$\begin{array}{c} v_{k|k-1}\left(x\right)=v_{S,k|k-1}\left(x\right)+\gamma_{k}\left(x\right) \end{array}$$
  (14) $$\begin{array}{c} v_{S,k|k-1}\left(x\right)=p_{S,k}\sum_{j=1}^{J_{k-1}}w_{k-1}^{j}N\left(x;m_{S,k|k-1}^{j},P_{S,k|k-1}^{j}\right) \end{array}$$
  (15) $$\begin{array}{c} m_{S,k|k-1}^{j}=F_{k-1}m_{k-1}^{j} \end{array}$$
  (16) $$\begin{array}{c} P_{S,k|k-1}^{j}=Q_{k-1}+F_{k-1}P_{k-1}^{j}F_{k-1}^{T} \end{array}$$
- Update:
  $$\begin{array}{c} v_{k}\left(x\right)=\left(1-p_{D,k}\left(x\right)\right)v_{k|k-1}\left(x\right)+\sum_{z\in Z_{k}}v_{D,k}\left(x;z\right) \end{array}$$
  (17) $$\begin{array}{c} v_{k|k-1}\left(x\right)=\sum_{i=1}^{J_{k|k-1}}w_{k|k-1}^{i}N\left(x;m_{k|k-1}^{i},P_{k|k-1}^{i}\right) \end{array}$$
  (18) $$\begin{array}{c} v_{D,k}\left(x;z\right)=\sum_{j=1}^{J_{k|k-1}}w_{k}^{j}\left(z\right)N\left(x;m_{k|k}^{j},P_{k|k}^{j}\right) \end{array}$$
  (19) $$\begin{array}{c} w_{k}^{j}\left(z\right)=\frac{p_{D,k}w_{k|k-1}^{j}q_{k}^{j}\left(z\right)}{\kappa_{k}\left(z\right)+p_{D,k}\sum_{l=1}^{J_{k|k-1}}w_{k|k-1}^{l}q_{k}^{l}\left(z\right)} \end{array}$$
  (20) $$\begin{array}{c} q_{k}^{j}\left(z\right)=N\left(z;H_{k}m_{k|k}^{j},R_{k}+H_{k}P_{k|k-1}^{j}H_{K}^{T}\right) \end{array}$$
  (21) $$\begin{array}{c} m_{k|k}^{j}\left(z\right)=m_{k|k-1}^{j}+K_{k}^{j}\left(z-H_{k}m_{k|k-1}^{j}\right) \end{array}$$
  (22) $$\begin{array}{c} P_{k|k}^{j}=\left[I-K_{k}^{j}H_{k}\right]P_{k|k-1}^{j} \end{array}$$
  (23) $$\begin{array}{c} K_{k}^{j}=P_{k|k-1}^{j}H_{k}^{T}{\left(H_{k}P_{k|k-1}^{j}H_{k}^{T}+R_{k}\right)}^{-1} \end{array}$$

As shown by these equations, the GM-PHD provides a computationally-efficient mechanism to propagate the posterior PHDs of multi-target state $X_{k}$; though with the passage of time, the GM-PHD filter suffers from computation problems associated with the increasing number of such Gaussian components. In practice, this problem is dealt by carrying out special pruning procedures in a part of each recursion, which removes insignificant or negligible Gaussian components based on some pre-designed criterion.

### 2.2. The Labeled Multi-Bernoulli Filter

In addition to the GM-PHD filters, other RFS-based approximations of the multi-target Bayes filters include multi-Bernoulli filters and their various extensions. The idea of the multi-Bernoulli filter was first proposed by Mahler [10] proposing a novel multi-target multi-Bernoulli recursion as a tractable approximation to the recursive Bayes multi-target filter under low-clutter density scenarios, whereby a multi-Bernoulli RFS distribution propagates directly as an approximation to the posterior multi-target state $X_{k}$ recursively instead of posterior PHDs.

However, [11] shows analytically that Mahler’s multi-target multi-Bernoulli filter (MeMBer) overestimates the cardinality and proposes a new variant called the cardinality balanced multi-target multi-Bernoulli (CBMeMBer) filter. The CBMeMBer filter extracts the cardinality bias of the MeMBer filter in the update step and uses this to develop an unbiased update at the end of its recursion. Like the PHD/CPHD filters, [11] has provided closed-form GM-based solutions in the case of linear/Gaussian state-space models. For general non-linear/non-Gaussian considerations, SMC-based implementations have also been provided. Interested readers are encouraged to refer to [11] for a further conceptual understanding of multi-Bernoulli filters along with the detailed analysis of their prediction and filtering steps.

While multi-Bernoulli filters are not formulated to output target tracks, their generalization, referred to as the generalized labeled multi-Bernoulli (GLMB) filters [12], has been proposed to overcome this limitation. These filters rely on the notion of labeled RFSs for their working principles. We direct the readers to [12,13,14] for the large quantity mathematic formation of this approach. This paper mainly elaborates the implementation design.

## 3. System Design and Implementation

This section describes the main essence or the methodology in coming up with the overall design of the pedestrian tracking system. This is followed by a detailed overview of the techniques and strategies utilized in carrying out the implementation of the overall system.

### 3.1. System Design Modules

**Figure 1:**
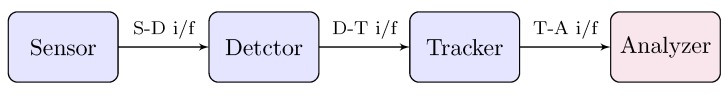
Block diagram of the multi-target tracking system.

Like any good engineering design, the main focus has been to come up with a modular design approach to overcome the system complexity efficiently, while aiding in quick development of the system with each module being designed in an isolated fashion and having a clear notion of its input/output interfaces. Figure 1 presents a high-level abstracted view of the overall pedestrian tracking system resulting from this approach. We explain these modules briefly as follows.

#### 3.1.1. Sensor

The sensor module represents an information-capturing device that extracts some useful target motion attributes (of interest) within the surveillance scene. This could be a stereo camera or a LiDAR sensor, etc., giving target motion information at regular intervals of time. Specifically for our work, we have made use of the following concepts as sensors:

- Simulated sensor model: For an extensive evaluation of the implemented pedestrian tracking system (Section 4), we design a simulation scenario simulating point targets whose motion follows linear/Gaussian characteristics. The specific simulation scenario can be considered as a form of sensor.
- Video frames: Likewise, we make use of MOT benchmark for further evaluation of the tracking system (Section 4). However, in this case, we are provided with camera video footage or frames comprising different kinds of pedestrian motions. These video frames then act as sensor observations.

#### 3.1.2. Detector

The detector module is responsible for extracting the target-specific information from the sensor outputs. Generally, this involves coming up with target approximate kinematic quantities from the sensor scans to feed into the tracker module. In our work, the detector detects the individual target’s 2D position coordinates within the surveillance region.

- Simulated detector model: For carrying out the simulation scenario, the simulated positions of the target from the sensor are corrupted with Gaussian noise to yield simulated detections. Furthermore, these detections are generated as part of the probabilistic process governed by a certain probability of detection $p_{d}$, which allows for target misdetections to help come up with robust tracker algorithms.
- Fast feature pyramid detector: The MOT benchmark provides detection annotations (2D position coordinates) on each of the training-video (sensor) frames. These detections are extracted by running the fast feature pyramid object detector algorithm, as proposed by Dollar et al. [15].
- Histogram of oriented gradients detector: The widely popular OpenCV library for developing computer-vision applications provides a working implementation of the HOG detector [16] targeting various platforms like C++, Python, CUDA, OpenCL, etc. We run this library function over MOT frames to come up with the target positions that are then fed into the tracker module further in the chain.

#### 3.1.3. Tracker

The tracker module is the most crucial/significant processing element of the overall system as it is responsible for outputting the target/pedestrian tracks utilizing the detections within every sensor-scan. As will be explained later, we have employed mainly two trackers in our work:

- GM-PHD tracker: This tracker implements the GM-PHD filtering recursions to estimate the 4D (2D position, 2D velocity) target state. The original GM-PHD filtering algorithm [1] is enhanced using the techniques presented in [17] in order to be able to extract not just individual target states, but rather their trajectories or tracks.
- SMC-LMB tracker: This tracker carries out the implementation of the SMC/particle-filter-based LMB filter [12,13]. The LMB filtering is based on labeled RFSs, which helps to extract target tracks from their states automatically. The implementation is carried out in C++, as well as using OpenCL acceleration.

#### 3.1.4. Analyzer

The analyzer module is the optional module responsible for analyzing the target tracks being produced from the tracker and compute various evaluation metrics enabling extensive evaluation of the implemented system (Section 4). Enabling this analysis, the analyzer module helps with coming up with stable efficient tracking system. Though, using this module could make sense in the development phase, it should be removed from the final system, as it provides no core functionality regarding pedestrian tracking.

#### 3.1.5. System Design Interfaces

The main intra-module interfaces carried out in the project as shown in Figure 1 are briefly described below:

- Sensor input: Generally, sensor input consists of the whole surveillance view/region containing targets of interest. For our project, this represents either a simulated scenario or an MOT tracking scenario.
- Sensor-detector i/f: The interface between the sensor and the detector mainly represents the sensor outputs. Further processing tasks could be carried out as part of this interface for helping the detector in its algorithm. However, as part of this project, we simply forward the sensor output frames into the detector. The frames are structured as 2D pixel data embedding the target motion information.
- Detector-tracker i/f: This interface mainly represents the target detections, which act as input stimuli to the target algorithm. For our work, these detections are in the form of 2D position coordinates (i.e., in the form of a 2D floating-point vector).
- Tracker output: This primarily represents the overall output of the whole system. In this work, output involves individual target 4D states (a 4D floating-point vector) along with a specific label (a two-integer structure) being output from the tracker module within every sensor scan.

#### 3.1.6. System Upgrades

As stated earlier, the proposed modular design methodology serves well to employ a plug and play-based design approach whereby one can easily replace an existing module for a better alternative without having to redesign the whole system from scratch, thus helping with efficient upgrading of the overall system. Some of the key upgrades in the present pedestrian tracking system could be:

- Sensor: There is no any specific requirement on the sensor. It can be a camera or a LiDAR. This would generate real-world sensor data for using the pedestrian tracking system in actual automotive scenarios to evaluate its effectiveness in carrying out its functionality.
- Detector: coming up with a detector algorithm of our own. This implemented detector would then help to do detections on the real scenario video footage.
- Tracker: further optimization of the implemented tracking algorithm, improving both the tracker accuracy, as well as its execution performance.

### 3.2. System Implementation

This subsection describes the major implementation aspect. As mentioned above, the sensor and detector are either simulated or are used from the MOT benchmark. The optional analyzer module will be discussed at length in Section 4. Therefore, this subsection mainly focuses on the implementation of the tracker module.

We initially carried out the implementation of the GM-PHD algorithm, which lacked the ability to output target trajectories. This was overcome by using a tree-based approach to group the GM-terms of a single target together to provide a notion of its trajectory. After extensive simulation analysis (Section 4), we found the GM-PHD filter accuracy to be inadequate in dealing with general pedestrian tracking scenarios where the pedestrians deviated from linear/Gaussian motion characteristics. This led to the exploration of SMC approaches to offer better accuracy. In light of this, a particle-filter implementation for the LMB filter was successfully carried out. Later via extensive profiling, the LMB filter implementation in C++ was accelerated via OpenCL kernels by spotting the performance bottlenecks and re-implementing them using parallel programming constructs.

#### 3.2.1. GM-PHD Tracker

**Figure 2:**
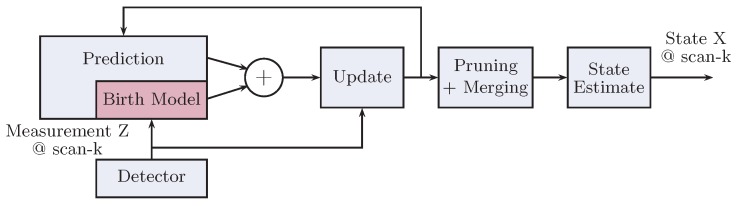
Block diagram of GM-probability hypothesis density (PHD) filter recursion.

The GM-PHD filter works by propagating the posterior PHD of the multi-target state in time during each of its recursions [1,17]. The GM-PHD filter recursion is carried out as shown in Figure 2. Each of the block represents a C++ class method performing its specific functionality. The arrows represent the data flow, whereby the GM terms representing the PHDs of multi-target state travel back and forth between the prediction and the update modules. Each of the Gaussian terms used throughout the filtering operation are compactly represented as a C++ struct with weight, mean and covariance as its attributes. To carry out the linear-algebra matrix operations in C++, we make use of efficient open-source library Armadillo. Therefore, while the weight is represented as a C++ float variable, the mean and covariance are better represented via armadilloclasses.

At the start of each filter iteration/recursion, the new scan detections are used by the birth model, which compares them to the stored previous-scan detections. Based on likely association or similarities between a specific pair within these consecutive scans, the birth model forms new targets by assigning a new set of Gaussian terms, i.e., a Gaussian mixture as part of the predicted GM. These components are then mixed/added with the predicted GM of the surviving targets (surviving targets are represented by update GM in past iteration) obtained via (15) and (16).

Similarly in the update block, the current-scan detections are used along with the current computed prediction GM to extract the update GM using (21) and (22). These Update GM terms are then passed through the prune/merge block where we use a three-fold strategy to reduce the computational complexity arising from the increasing GM terms. These are summarized as follows:

- First, all of the close-by Gaussian terms (via their means) are merged together to form a composite Gaussian term, as they are thought to represent a single target.
- Then, all of the Gaussian terms whose weights are less than the filter-specified threshold are discarded as insignificant terms and are not processed further. This also allows one to gracefully terminate target tracks.
- Finally, we keep a cap on the maximum number of Gaussian terms corresponding to the maximum number of expected pedestrians within the surveillance zone.

After the pruning, the update GM represents the posterior PHD of the multi-target state with each Gaussian term representing a possible target state. To avoid tracking clutter terms, a second threshold is used here to discard the Gaussian terms that are not too significant as of the current iteration, but could lead to greater weights in coming iterations. Such terms are not output as current target states, but also not discarded, as they are being kept in the surviving target GM to be considered for the next iterations. For the significant terms, their means represent the individual target states and are output as such.

As should be obvious from these outputs, this preliminary filter is only capable of extracting individual target states, but does not output tracks or trajectories, i.e., there is no association between currently obtained states and the past ones. To overcome this limitation, the implementation algorithm is enhanced via tree-based techniques. For the sake of brevity, we ignore here the implementation details of those techniques, however. Interested readers are referred to [17] for further details in this regard.

#### 3.2.2. SMC-LMB Tracker

The GM-PHD tracker provides the optimal PHD recursive solution in the case of targets and sensors following linear/Gaussian state-space characterizations. Therefore, expectedly, its accuracy performance should be deteriorated to a certain degree when the targets exhibit non-linear and/or non-Gaussian motion tendencies. We carry out simulation analysis (Section 4) to investigate this and find that the tracker performance is severely affected up to a point that the error is too much to tolerate. This led to the exploration of non-linear techniques to overcome this limitation.

SMC-based particle filters have been a popular approach in this context after being introduced in the 1990s. We looked into the possibility of using the particle filter implementation of the PHD filter in order to make it suitable for tackling generic pedestrian motions. However, we found that, recently, a new class of RFS-based filters called the LMB filters has been proposed, which are deemed to be more accurate than PHD filtering techniques. Furthermore, the SMC implementations of these filters match with those of PHD filters in terms of computational complexity [14]. Therefore, this work further focuses on implementing the SMC-LMB tracker as the main tracker module in Figure 1.

**Figure 3:**
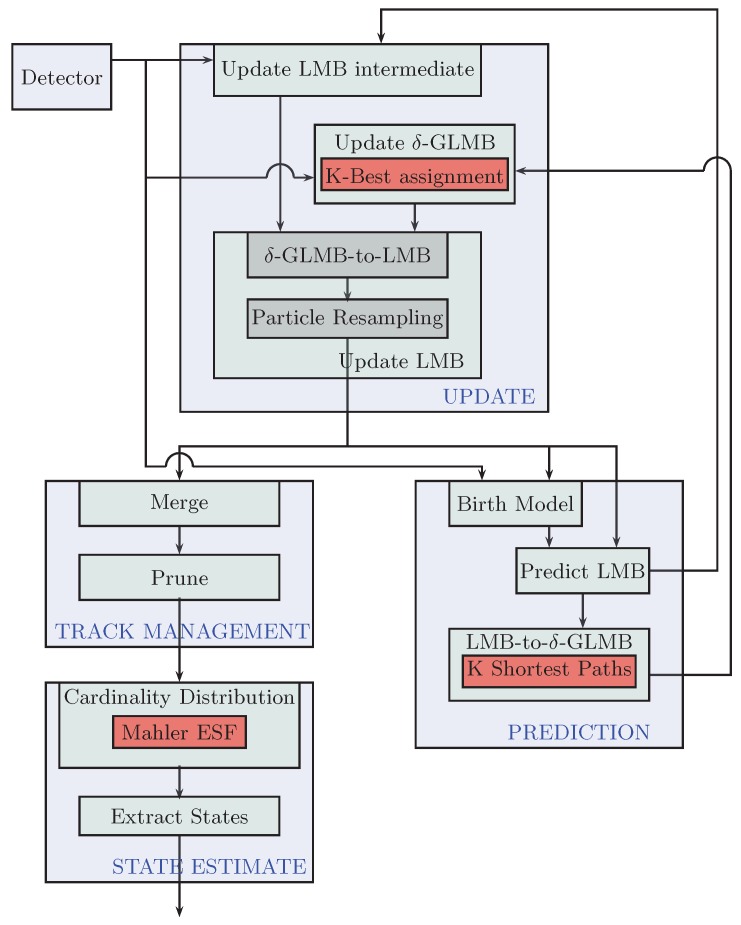
Block diagram of sequential Monte Carlo (SMC)-labeled multi-Bernoulli (LMB) tracker recursion.

Figure 3 presents the overview of the implementation of the SMC-LMB tracker. Structurally, it is similar to Figure 2, like any Bayesian estimator, but computationally, there are major differences. First, instead of using GM to represent the posterior states, the SMC-LMB tracker relies on propagating a set of multi-Bernoulli terms. Similar to the GM-PHD filter implementation, these terms are compactly represented as a C++ struct using standard floats while the 4D state is conveniently represented via Armadillo vectors. Moreover, based on labeled RFSs, each instance of this struct has a unique 2D intlabel vector that acts as a tag for each tracked target. Each of the sub-blocks shown in Figure 3 is implemented via C++ functions as member functions of the LMB filter class.

- Filter initialization: This function is executed once for each instance of the tracker class at the time of its construction. Here, all of the tracker parameters are set, like the number of particles to represent state-pdfs, the maximum number of LMB components allowed to represent the posterior multi-target state, etc., along with the state-space modeling parameters.
- Birth model: Similar to GM-PHD filter implementation, the birth model in the SMC-LMB tracker implementation relies on the associations between the measurements obtained in consecutive scans. However, in the case of the birth of new targets, instead of representing it via a GM, a new multi-Bernoulli term is generated. In the current implementation, we find speeds in the Cartesian space between every detection pair using the current and the immediate past detection scan. If the speeds for a specific pair lie within the tracker-parametrized $V_{max}$ value, the pair is deemed to correspond to a single target, and hence, a new target track is created. This track is initialized via parametrized existence probability while its state-pdf is supposed to be a Gaussian, and a certain number of particles are drawn from it stochastically. This number of particles is also parametrized, and we recommend them to be within powers of two for ease in GPU particle-level processing.
- Predict LMB: This module carries out the LMB prediction. Specifically, it generates predict-LMB terms for new-born targets from the birth model, as well as survive-LMB terms for existing targets via current update-LMB terms. We use two different sets of LMB terms instead of a single one, as it is much easier to do further conversion into δ-GLMB components separately and then merge them together.
- Predict-LMB to predict-δ-GLMB: Both of computed birth predict-LMB, as well as surviving predict-LMB terms are then converted to their δ-GLMB terms. This step is necessary to allow the δ-GLMB update later in the data flow. Now, even for a moderate number of targets/pedestrians, these δ-GLMB could become large, and processing them quickly becomes computationally expensive. Like for the case of the GM-PHD tracker, we introduce pruning schemes to cap the maximum number of components. However, in contrast to the former approach, the components have not yet been computed. Therefore, to avoid computation of all such components followed by the propagation of significant components, we rather formulate this problem as a K-shortest path problem and use the computationally-efficient Eppstein solution [18] (using the Bellman–Ford algorithm [19] internally) to directly compute only the significant components without the need for further pruning. Interested readers are encouraged to read [4] in order to come up with such a formulation.
  After computing the separate δ-GLMB components for the new-born and existing targets, they are convolved together to give the overall δ-GLMB terms that are used for the update phase in the next scan/iteration while the LMB terms are simply concatenated together.
- Update LMB intermediate: This is the first step within the update step of the SMC-LMB recursion. It computes all possible update LMB terms based on every possible association of the current measurement-set with the previous scan predict LMB terms. These terms would be required later on in conversion of update δ-GLMB terms to their equivalent LMB terms. Hence, these terms are considered intermediate within the recursion.
- δ-GLMB update: This step performs the closed-form δ-GLMB update on the predict δ-GLMB [4] terms as obtained in the previous iteration. Here, again, we are confronted with the similar problems of rapidly growing terms and have to employ some sort of a cap on the maximum number of components to deal with computational complexity. However, because of the measurement involvement, this problem is formulated as the K-best assignment problem as opposed to the K-shortest path problem. To solve this problem, we rely on using the Murty algorithm [20] (using the Hungarian method internally) as explained in greater detail in [4].
- Update LMB: As shown in Figure 3, coming up with the update LMB terms within each tracker iteration involves a two-fold process. First, a conversion from update δ-GLMB terms to corresponding LMB terms is carried out such that the LMB set matches the PHD terms of the δ-GLMB set as was explained in [13]. Next, the particles needed to represent each of LMB term’s state probability density functions $p^{i}\left(.\right)$ are replaced with new set in a commonly-used procedure referred to as particle resampling to deal with the particle impoverishment problem. The computation of these LMB components completes the SMC-LMB recursion.
- Track management: In contrast to GM-PHD tracker, no special procedures are required to output target tracks, as the SMC-LMB filter outputs update LMB terms containing unique tags, i.e., outputting target tracks or trajectories directly. Here, again, the techniques of merging (to combine tracks formed from single target) and pruning (for tracks that we are not yet confident of being either a new target or clutter) are used just like for the case of the GM-PHD tracker.
- State estimation: The final step within each tracker iteration is to estimate individual target states and to associate them to already existing tracks/trajectories. For this, we use Mahler’s ESFfunction [4] to first estimate stochastically the cardinality of the current multi-target state based on the pruned update LMB terms. Then, a certain number of most weighted/significant components corresponding to this cardinality estimate is chosen for state-estimation. Using the particle representation of these components, an empirical measure is easily derived for each chosen component.

### 3.3. OpenCL Acceleration

Being satisfied with the tracking accuracy of the SMC-LMB tracker (Section 4), an extensive profiling of the above-mentioned algorithm in C++ was carried out for the purpose of spotting potential performance bottlenecks. As clear from the detailed analysis presented in Section 4, the primary source of execution performance bottleneck within the recursion is the computation of update LMB terms, roughly amounting to 75% of the computations. Therefore, in our strategy to improve the execution performance of the algorithm, instead of redesigning the whole algorithm from scratch via programming constructs, we relied rather on a hybrid of C++ and OpenCL computation code. We transformed the sequential execution of the update LMB function into OpenCL kernels to significantly improve the timing performance of the whole algorithm. The main implementation aspects of this strategy are outlined below:

- Generation of uniform random numbers on the GPU itself using the AMD CLRNGcompute library.
- Breaking down the for-loops within the update block down to the level of particle computations.
- Efficient parallel scan (prefix-sum) on the cumulative weight array to carry out the particle resampling procedure.
- Optimized memory organization for the LMB terms throughout the update part of the recursion as a high amount of memory transfers between the host CPU and GPU accelerator severely affects the performance and could possibly outdo the benefits achieved via GPU computations.
- The number of particles allocated for each of the LMB terms is chosen to be in powers of two, which makes it easier to use shared-memory optimizations within GPU computations for further acceleration of the application.
- We use the extensive vector operation module for vectorizing code.
- Each Gaussian component is computed in a separate thread; as the number of targets increases, the number of components increases very rapidly. This feature exploits all advantages of a parallel architecture.

## 4. Experimental Evaluations

This section presents a detailed evaluation and analysis of the pedestrian tracking system as explained in previous sections. Primarily, the analysis was carried out in two-fold. We discuss the tracking accuracy performance of the proposed design followed by its execution performance analysis.

### 4.1. Evaluation Metrics

It is paramount to have a clear notion of evaluation metrics before carrying out the actual evaluation of the system itself. Given below is a brief overview of these evaluation metrics, which will be used to evaluate the proposed system.

#### 4.1.1. Tracking Accuracy

As mentioned earlier, the multi-target tracking problem attempts to jointly find the number of targets, as well as their individual states from the received measurements with the passage of time. Therefore, to quantify such a tracker’s accuracy, we make use of following metrics:

- Cardinality estimate: this metric extracts the number of targets at the end of each tracker recursion. This can then be compared with the truth/actual cardinality of the multi-target state to figure out the tracking errors in this respect.
- Optimal sub-pattern assignment (OSPA): this metric defines a notion of mis-distance and corresponding error between actual and estimated individual target states as proposed firstly by [21].

#### 4.1.2. Execution Performance

For profiling the tracker algorithm’s execution performance, we use a simple mechanism involving the computation of the number of CPU cycles across the algorithm. In the context of video processing, we can obtain an average measure of frames per second as a computational throughput measure for the tracker via:

(24) $$fps=\frac{\mathrm{Number}\;\mathrm{of}\;\mathrm{frames}\;\mathrm{in}\;\mathrm{video}}{\mathrm{Total}\;\mathrm{execution}\;\mathrm{time}}$$

### 4.2. Tracking Accuracy Analysis

This section details this work’s findings regarding the tracking performance of the proposed LMB tracker. In order to evaluate the tracker for providing this analysis, we make use of a simulation analysis, as well as using available dataset videos.

#### 4.2.1. GM-PHD and SMC-LMB Comparisons

For carrying out an extensive analysis for evaluating the tracking accuracy of the implemented LMB tracker, we created a 2D surveillance point-target-based simulation scenario. The target motion dynamics, as well as the sensor model were made to follow linear/Gaussian characteristics. Apart from that, the simulation was designed to be highly parameterized in the number of targets; their birth locations; their birth times and death times; their detection and survival probabilities; the amount of clutter; process/measurement noise, etc. This helps to generate a diverse range of simulation scenarios to adequately understand the tracking accuracy performance under the influence of different constraints.

For the comparison, we designed two artificial simulation scenarios, whereby we simulated 12 linearly moving targets for a duration of 100 time steps. The first scenario was designed to show the accuracy performance of the algorithm under highly ideal tracking conditions, while the other scenario posed slightly more challenging conditions. These were parameterized as:

- Easy tracking scenario: $p_{S,k}=0.98,\;p_{D,k}=0.98,\;\sigma_{v}=1\;m/\sec^{2},\;\sigma_{w}=1\;m,\;▵=1\;s,\;\lambda_{c}=5$.
- Hard tracking scenario: $p_{S,k}=0.90,\;p_{D,k}=0.90,\;\sigma_{v}=5\;m/\sec^{2},\;\sigma_{w}=10\;m,\;▵=1\;s,\;\lambda_{c}=60$.

where $p_{S,k},\;p_{D,k}$ are the survival and detection probabilities. ▵ is the sensor sampling period or the inter scan-time. $\sigma_{v},\sigma_{w}$ represent the variances within the Gaussian process and measurement noises, respectively. The parameter $\lambda_{c}$ denotes the clutter density, which is defined to be the average number of clutter returns.

**Figure 4:**
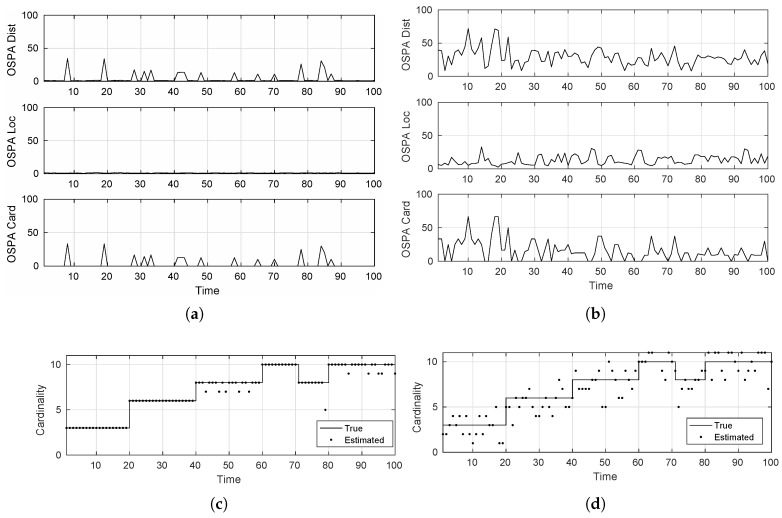
Tracking accuracy of the GM-PHD tracker. (**a**) Optimal sub-pattern assignment (OSPA) distance (easy-scenario); (**b**) OSPA distance (hard-scenario); (**c**) cardinality estimate (easy-scenario); (**d**) cardinality estimate (hard-scenario).

As can be seen from Figure 4, the GM-PHD tracker performs apparently perfectly in the easy scenario for estimating the individual target states as illustrated by the OSPA measure. The cardinality estimate, however, shows that even in ideal conditions, the tracker does make occasional mistakes. This is attributed to the adaptive birth distribution model, being embedded inside the tracker algorithm, which requires some initial scans to confirm successive measurements of the new target in order to confirm it as a new track. This leads to cardinality errors at target births. Avoiding this can lead to tracker performance being severely affected, causing it to consider every new detection as a new target, which could very well be a clutter detection or a false-alarm. Similarly, when the target dies, the tracker expectedly makes mistakes because the algorithm cannot be sure about the target disappearance as an actual death or a miss-detection because of sensor imperfection. If one designs the tracker algorithm to abruptly terminate tracks just because of one misdetection, then tracker performance could suffer drastically. Consider for example the occurrence of a target misdetection. When such a target is re-detected, then the tracker would consider it to be a new target/track instead of continuing the previous known track. This behavior in most tracking scenarios is undesirable. Furthermore, these plots clearly show the worsening performance of the tracker in dealing with more severe tracking environments.

**Figure 5:**
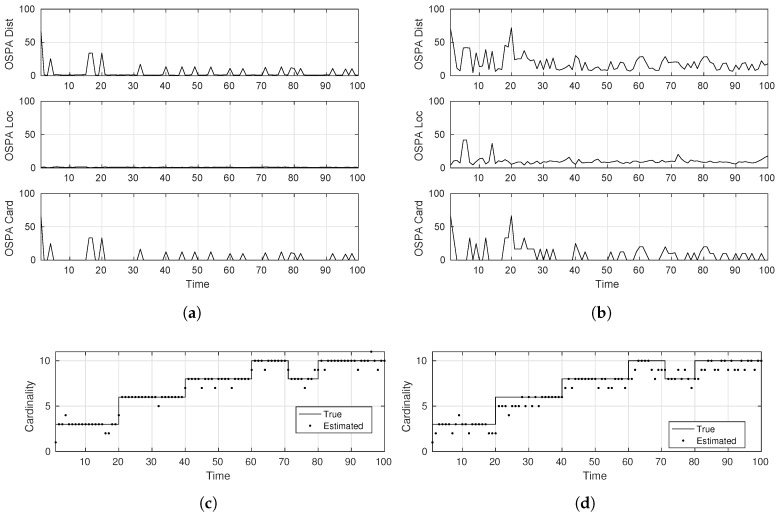
Tracking accuracy of the SMC-LMB tracker. (**a**) OSPA distance (easy-scenario); (**b**) OSPA distance (hard-scenario); (**c**) cardinality estimate (easy-scenario); (**d**) cardinality estimate (hard-scenario).

To evaluate the SMC-LMB tracker, we used a configuration of 512 particles and cap the update δ-GLMB hypotheses and the δ-GLMB components to a maximum of 100. Figure 5 presents the corresponding results under the designed simulation scenarios. It is a clear from these plots that using a sufficiently high number of particles/samples (like 512 in our case) to approximate the true posterior density, the LMB tracker shows comparable, if not better, performance than the more suitable GM-PHD tracker for linear/Gaussian systems. Especially under severe tracking scenarios where the targets show considerable deviation from the linear/Gaussian as governed by the higher $\sigma_{v}$ value, these results justify the use of SMC-based trackers for MTT tracking, by showing their robustness in dealing with process modeling imperfections.

#### 4.2.2. MOT Dataset Analysis

This subsection presents the tracking accuracy performance of the LMB tracker system on publicly available dataset videos. The goal of the analysis is to further build on the understanding of the tracking accuracy analysis developed via comprehensive simulations and to see whether the simulation results correspond to pedestrian tracking scenarios in actual video footage.

For this purpose, we use an effective benchmark named the Multiple Object Tracking Challenge [3]. This benchmark is designed to provide video footage covering diverse multi-target tracking contexts where the goal is to track pedestrians accurately. The benchmark is designed as a competition where the current state-of-the-art approaches are ranked as per their tracking accuracy. The pedestrian detections are already provided by the benchmark so the accuracy of tracking directly depicts the efficiency of the tracking algorithm in dealing with various tracking challenges.

For the sake of brevity, in this section, we present an evaluation analysis of the LMB tracker on two of the MOT videos as summarized in Table 1:

- KITTI-17: static camera; mostly linear target motion
- PETS09-S2L1 static camera; targets move in irregular patterns

**Figure 6:**
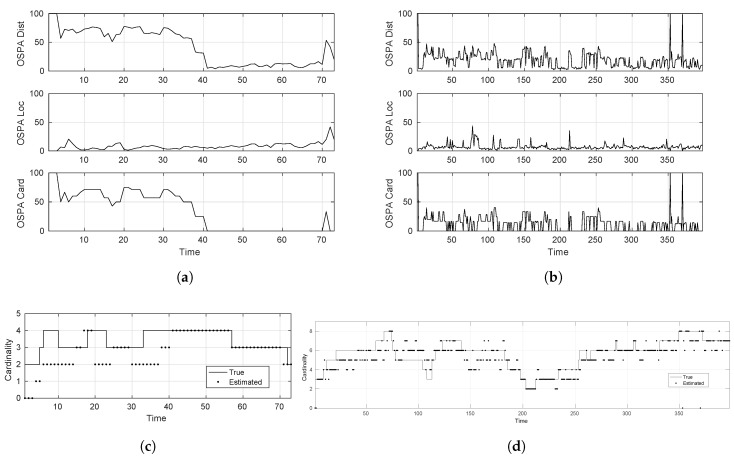
LMB tracker accuracy: MOT dataset videos. (**a**) OSPA distance KITTI-17; (**b**) OSPA distance PETS09-S2L1; (**c**) cardinality estimate KITTI-17; (**d**) cardinality estimate PETS09-S2L1.

Figure 6 shows the corresponding accuracy plots when these videos are fed to the LMB tracker for pedestrian tracking. For the KITTI-17 video, the tracker takes some initial frames to confirm target tracks, thereby making cardinality errors in the initial frames, but once the tracks are confirmed, the tracker performs highly accurately in tracking each of the pedestrian motions. The reason for such small OSPA distances in the later phase of the video can be attributed to the motion characteristics of the pedestrians. In the KITTI-17 video, most targets move in a straight line, i.e., in a linear fashion, hence the tracker performs as expected, as well as per the simulation analysis.

On the other hand, the PETS09-S2L1video presents a much tougher challenge in that the pedestrians move in irregular patterns like moving abruptly or moving in circles, etc. As mentioned earlier, due to the use of linear state-space models internally in the current SMC-LMB tracker implementation, the accuracy performance deteriorates. Specifically, due to model deviation from actual pedestrian motion, the tracker keeps on making erroneous predictions, and when the corresponding target’s measurement does not tally with this prediction, the algorithm terminates the track as evident by regular cardinality errors in the plots. Furthermore, the OSPA distances are relatively high as compared to the KITTI-17 plots.

#### 4.2.3. Execution Results:

In a heterogeneous platform with an Intel Xeon processor and an AMD GPU W7100, we carried out the extensive profiling of the implemented SMC-LMB tracker algorithm. First, we extensively evaluated the execution performance of the C++ code to spot the potential performance bottlenecks. These were then subjected to OpenCL acceleration to come up with an implementation meeting the real-time constraints. We made use of the MOT videos (Table 1) to carry out this analysis.

For helping in spotting the potential bottlenecks, we split the main C++ algorithm into six parts, which are: (a) predict LMB; (b) predict δ-GLMB; (c) update LMB intermediate; (d) update δ-GLMB; (e) update LMB; (f) track-management and state-estimation.

**Figure 7:**
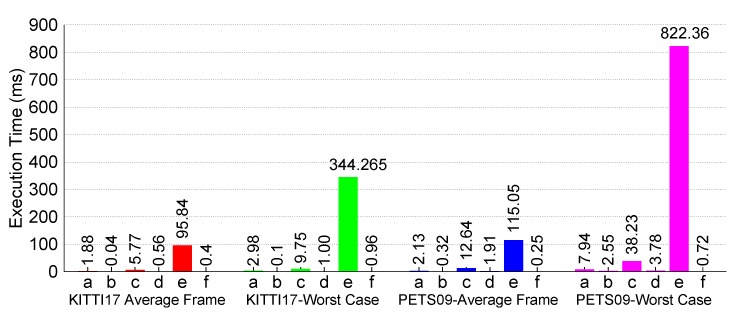
C++ LMB tracker execution performance.

Using the CPU clock-cycle metric as mentioned at the start of this section, we present our findings in Figure 7. These plots clearly show that the update LMB computation is the major bottleneck within the sequential SMC-LMB implementation. For KITTI-17, we get an average fps of 20 fps, while for the worst-case (WC) frame, we get up to 5 fps. For a more tough video PETS09-S2L1, we rather get an average fps of 15 fps over all frames, while the WC frame corresponds to about 2 fps.

**Figure 8:**
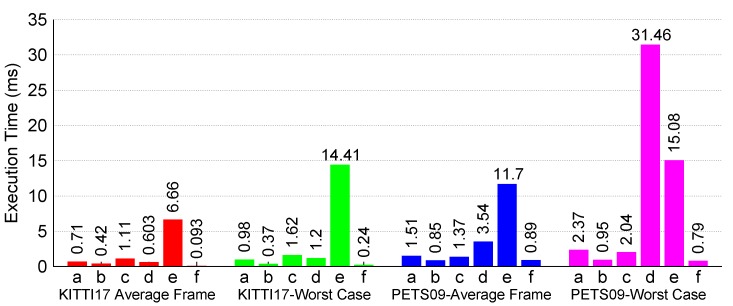
OpenCL accelerated LMB tracker execution performance.

**Figure 9:**
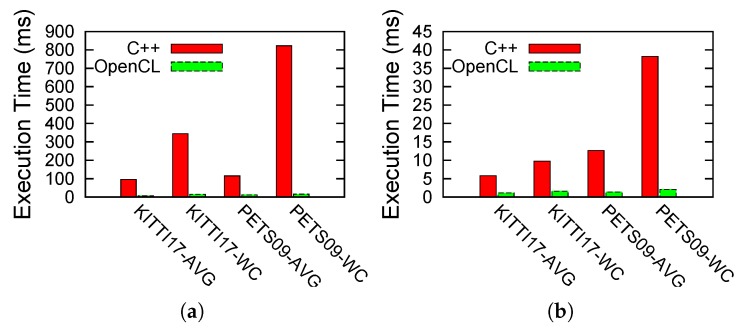
Comparison between C++ and OpenCL LMB implementations. (**a**) Update LMB computation, (**b**) Update LMB intermediate computation.

As explained in Section 3, we target the update LMB computation using OpenCL compute kernels. Using this acceleration, we manage to gain substantial improvements in terms of execution performance, as shown in Figure 8, where for the PETS09-S2L1 video, we now get an average fps of 100 fps, while the WC frame amounts to 36 fps. Figure 9 further compares the OpenCL timings directly with the C++ ones to further show the substantial gains in performance for the two algorithmic functions that have been accelerated up till now.

**Figure 10:**
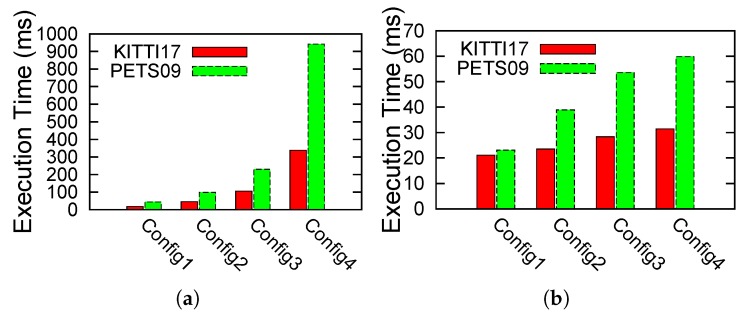
Scalability of C++ and OpenCL LMB implementations. (**a**) C++ implementation; (**b**) OpenCL implementation.

Finally, we also carried out the profiling of the LMB tracker algorithm under its different configurations. We studied the impact of changing its number of particles, birth components, survive components and update components. For this purpose, we used four different configurations (Table 2) to tackle a variant of the hard-scenario using $\lambda_{c}=5$. These results are presented in Figure 10. As shown in these plots, the OpenCL implementation not only offers advantages in terms of sheer execution timings, but also provides a much more scalable implementation as compared to its pure C++ counterpart. The execution times rise nearly exponentially for both MOT videos in the case of the C++ version, while the rise is much less steep or rather linear for the case of the OpenCL-based implementation.

At last, we have to note that there is still much room to improve the computation time while maintaining the tracking accuracy. In this paper, the prediction and update have been processed in two separate steps. This separation decreases the computation efficiency. In a recent paper, Vo et al. [5] proposed an efficient implementation by combining the prediction and update into a single step, which has a linear complexity in the number of hypothesized objects. It has been shown in [5] that the joint computation can speed up the execution time ranging from a dozen times to a thousand times for the linear Gaussian scenario. In our scenario, the joint computation can improve the performance on KITTI17 more than on PETS09 because most targets in KITTI17 move linearly. Figure 8 shows that the computation part of updating and predicting has taken 99% of the computation for KITTI17 and 95% for PETS09. It is therefore estimated that that there will around a 10- to 100-times computation speedup. The speedup effect on PETS09 will be less than on KITTI17 because targets in PETS09 move in a nonlinear way.

## 5. Conclusions

In this paper, we have developed two random finite set-based Bayesian filtering approaches, Gaussian mixture probability hypothesis density (GM-PHD) and labeled multi-Bernoulli (LMB) filters. The two approaches were designed in a highly modular way. After conducting their accuracy evaluations towards the multi-target tracking problem, we found that LMB filters were more appropriate to track the pedestrians. Then, we implemented in the LMB filter in C++ and carried out an extensive execution profiling. OpenCL programming was used to relieve the execution burden from the execution bottlenecks. The experimental results demonstrated a high computation improvement. In particular, the frame per second was improved from 15 fps to 100 fps on average, and the worst-case computation was also improved 18× from 2 fps to 36 fps.

## Figures and Tables

**Table 1:** MOT dataset videos.

Video	Resolution	Number of Frames	Unique Targets	Maximum Targets per Frame	Target Density
KITTI-17	1224 × 370	145	9	4	4.7
PETS09-S2L1	768 × 576	795	19	8	5.6

**Table 2:** LMB tracker configurations.

# Particles	# Births	# Survivals	# Updates
128	5	20	20
256	10	50	50
512	20	100	100
1024	50	200	200
